# Venetoclax in combination with chidamide and azacitidine for the treatment of relapsed/refractory B-cell acute lymphoblastic leukemia with the *MLL-AF4* gene: a case report and literature review

**DOI:** 10.3389/fimmu.2024.1475974

**Published:** 2025-01-14

**Authors:** Xuelian Jin, Zhigang Liu, Yu Wu, Jie Ji

**Affiliations:** Department of Hematology, West China Hospital, Sichuan University, Chengdu, Sichuan, China

**Keywords:** relapsed/refractory, *MLL-AF4*, ALL, leukemia, Bcl-2 inhibitor (venetoclax), histone deacetylase inhibitor (chidamide), demethylation (azacitidine), targeted therapy

## Abstract

B-cell acute lymphoblastic leukemia (B-ALL) with the *MLL-AF4* fusion gene has a poor prognosis, and the mortality rate exceeds 90%, particularly in cases of extramedullary relapse (EMR). Herein, we present a case of a 46-year-old male patient who developed relapsed B-ALL with *MLL-AF4*. The patient initially achieved a complete remission (CR) after induction therapy and underwent haploidentical hematopoietic stem cell transplantation. Five months post-transplantation, he developed enlarged lymph nodes and subcutaneous masses. A lymph node biopsy confirmed EMR, without leukemia in the bone marrow or peripheral blood. The patient received the VCA regimen (venetoclax, chidamide, and azacitidine) and was regularly monitored through blood counts, marrow cell morphology analysis, flow cytometry, and computed tomography or positron emission tomography-computed tomography imaging. After the first VCA course, the patient achieved a second CR with only transient myelosuppression. Following two VCA courses, he received chimeric antigen receptor T-cell therapy, which led to complete metabolic remission and improved prognosis. This case underscores the potential of the VCA regimen as a bridging therapy for EMR in B-ALL with *MLL-AF4*, although further studies are warranted.

## 1 Introduction

In adult patients with B-cell acute lymphoblastic leukemia (B-ALL), induction therapy can typically exceed a complete remission (CR) rate of 90%. However, the long-term survival is only 30–60% among those treated with a standard chemotherapy with or without allogeneic hematopoietic cell transplantation (allo-HCT). Moreover, relapse is commonly reported in 25–30% of patients who have undergone allo-HCT (1–4) plausibly due to unfavorable cytogenetic changes such as *MLL* rearrangements (MLL-r) with t(4;11)(q21;q23) that results in the formation of the *MLL-AF4* gene (also known as *KMT2A-AFF1*), which is associated with poor prognosis.

Research has revealed B-cell lymphoma-2 (Bcl-2) overexpression, histone deacetylation, and extensive promoter hypermethylation in cells among patients with MLL-r. We hypothesize that combining therapeutic agents targeting these three major pathogenic mechanisms will control B-ALL with MLL-r, especially in relapsed and refractory (R/R) patients. This report presents a case of B-ALL with MLL-r who developed extramedullary relapse (EMR) after undergoing allo-HCT, and was treated with the VCA regimen—a combination of venetoclax (a Bcl-2 inhibitor), chidamide (a selective histone deacetylase inhibitor), and azacitidine (a DNA demethylating agent)—which resulted in a CR.

## 2 Case description

A 46-year-old male patient presented with multiple enlarged lymph nodes throughout his body in June 2023. According to results of blood tests, bone marrow (BM) aspiration, and other relevant examinations, the patient was diagnosed with acute pre-B-cell lymphoblastic leukemia (Pro-B-ALL) with *MLL-AF4*. Chromosome karyotyping analysis revealed 45,XY,t(4;11) (q21;q23),add(5)(p15.3),der(5)t(5;12)(p15.3;q13),12 (5)/46,XY (11) (classified as the poor prognosis group as per NCCN 2021 guidelines) (5). The patient received an induction with one course of VDLP (vindesine, daunorubicin, L-asparaginase, and prednisone). Flow cytometry examinations following induction treatment revealed morphologic CR with 2.15×10^−2^ minimal residual disease (MRD). Since August 2023, the patient received one course of blinatumomab (35 µg ×9) as a consolidation therapy. Reexamination of the BM following consolidation therapy revealed CR with negative MRD. In September 2023, the patient with the help of his sibling sister as the donor underwent haploidentical hematopoietic stem cell transplantation with myeloablative conditioning and PTCy-based graft-versus-host disease (GvHD) prophylaxis. The engraftment was successful, and there was no apparent GvHD. The patient remained in CR with negative MRD thereafter. Considering the poor prognosis of B-ALL patients with MLL-r, the patient received blinatumomab as maintenance therapy from 3 months post-transplantation as an infusion from 9 to 18 µg/day for 7 days.

**Figure 1:**
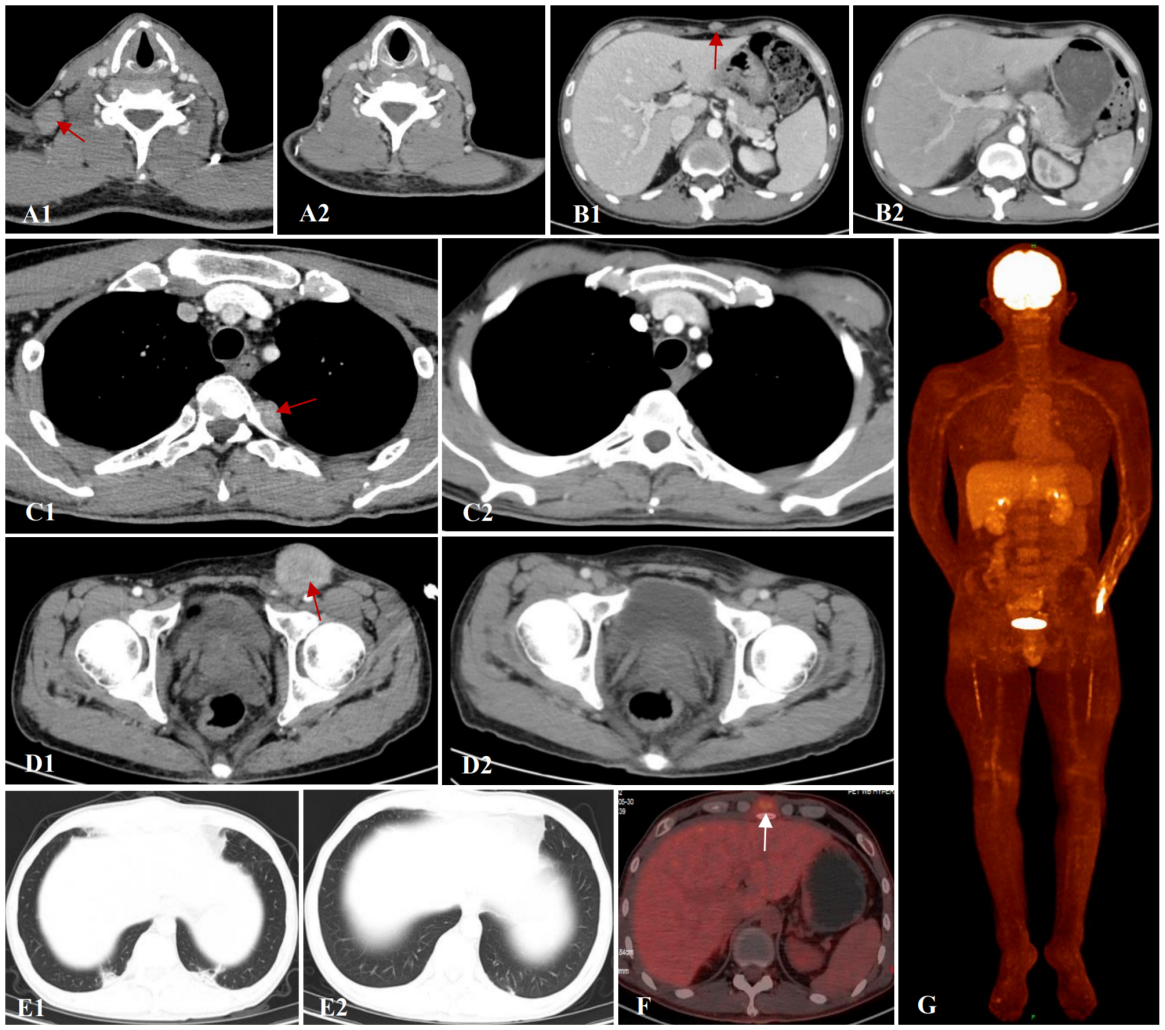
Contrast-enhanced CT of the neck, chest and abdomen. **(A1, A2)** Axillary lymph nodes before and after VCA treatment. **(B1, B2)** Soft tissue mass around the subxiphoid before and after VCA treatment. **(C1, C2)** Paravertebral soft tissue image before and after VCA treatment. **(D1, D2)** Soft tissue mass in the inguinal region before and after VCA treatment. **(E1, E2)** Pulmonary infection before and after antifungal therapy. **(F, G)** PET-CT revealed active ^18^F-FDG metabolism in the superficial fascia and xiphoid process of the right chest wall after VCA treatment.

In February 2024, 5 months post-transplantation, the patient complained of multiple enlarged lymph nodes and subcutaneous masses. A prompt biopsy of the left inguinal lymph node revealed consistent medium sized lymphocytes with a high nuclear−cytoplasmic ratio and a morphology resembling that of lymphoblasts. Immunohistochemical analysis revealed their characteristics as CD20^−^PAX-5^+^ CD19^+^ CD22^−^CD3^−^CD10^−^TdT^−^CD34^−^CD43^+^CD117^−^MPO^−^, Ki-67 (MIB-1) (+, 80–90%), and EBER1/2-ISH negative. Gene rearrangement analysis by polymerase chain reaction showed the presence of *IgH* gene clonal expansion in the target fragment range. Flow cytometry analysis of the same sample confirmed that 61.4% of nucleated cells were lymphocytes. Among them, B-cell population was approximately 61.8%, of which CD45^dim+^CD19^dim+^CD20^−^CD22^−^CD5^−^CD10^−^CD23^−^FMC7^−^CD38^+^Bcl-2 (+, in small amounts) TDT^−^HLA-DR^+^CD34^−^CD117^−^MPO^−^CD14^−^CD13^−^CD33^−^CD64^−^CD123^−^ and mlgK (−), mlgλ (−) confirmed EMR. However, there was no evidence of leukemia in the BM or peripheral blood sample. BM cells showed complete donor chimerism. Contrast-enhanced computed tomography (CT) of the neck, chest, and abdomen (**Figures 1A1, B1, C1, D1**) revealed the presence of enlarged lymph nodes in bilateral submandibular spaces, adjacent to the carotid sheath and within the posterior triangle of the neck, supraclavicular regions, and axilla. The largest node was in the left inguinal fossa and measured 4.1×4.0 cm. Soft tissues were observed, adjacent to the T4 vertebra on the left side, T7-8 vertebra on the right side, and beneath the sternum. In addition, soft tissue nodules were detected in local areas of the anterior superior abdominal wall and the chest wall below the right axilla, the largest nodule being approximately 1.7×1.1 cm. Moreover, patchy nodules with ground−glass opacities and diffused distribution were scattered in both lungs, the lower lobes of which showed subpleural consolidation in the posterior basal segment (**Figure 1E1**).

As the patient had relapsed early (within 6 months post-transplantation) from allo-SCT and blinatumomab maintenance, classical therapies such as chemotherapy or donor lymphocyte infusion may not be effective enough for remission reinduction. We recommended this patient to receive anti-CD19 chimeric antigen receptor T-cell (CAR-T) as salvage therapy. However, after his peripheral blood mononuclear cells were harvested, he experienced progressive enlargement of aforementioned lymph nodes and soft tissue masses, which necessitated an optimal bridging therapy.

**Figure 2:**
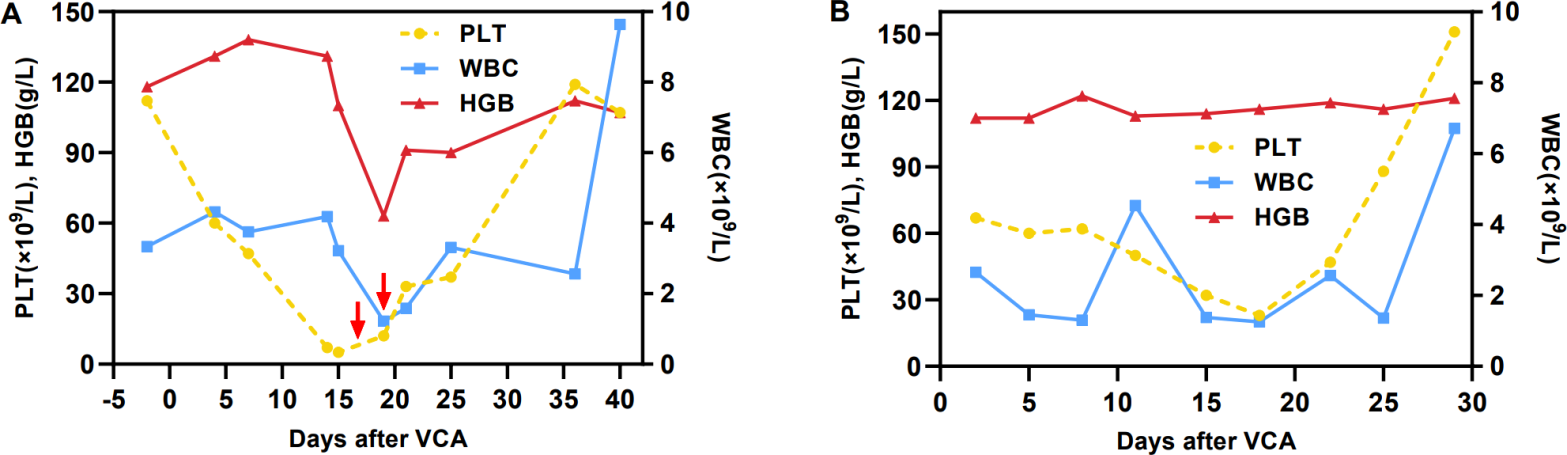
Hemoglobin, white blood cell and platelet counts. **(A)** First course, **(B)** second course. The red arrow indicates the infusion of one therapeutic dose of platelets.

**Figure 3:**
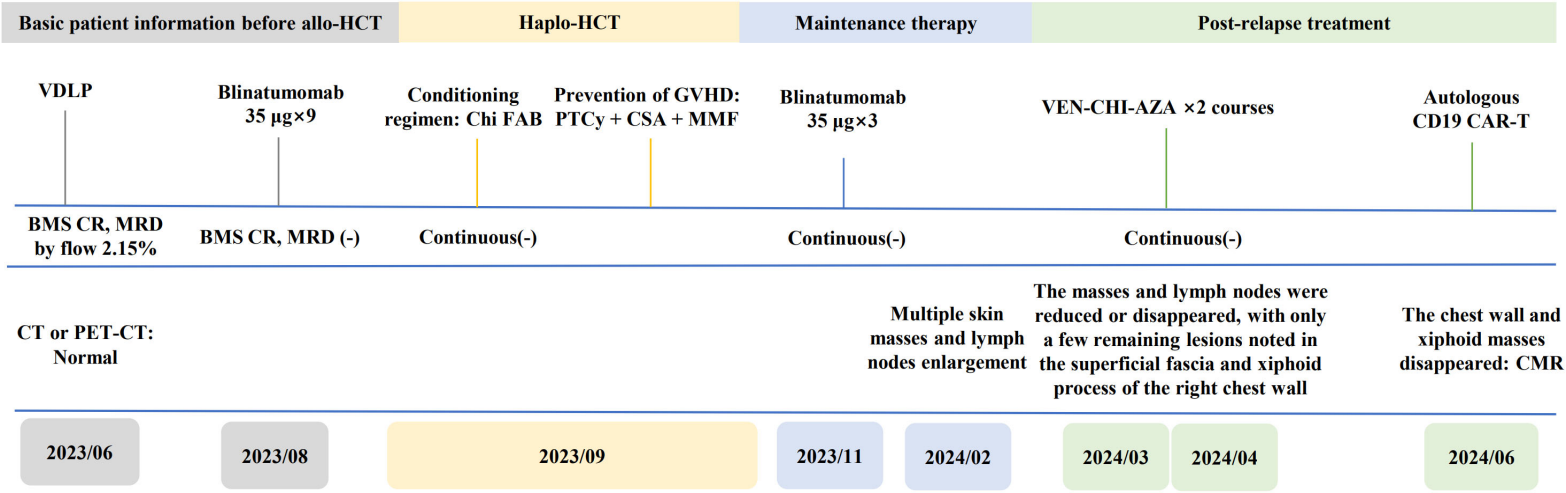
Timeline of the patient’s treatment and bone marrow, CT or PET-CT assessment. BMS, bone marrow smear.

On March 8, 2024, the patient received VCA regimen (venetoclax 200 mg daily on days 1-14, chidamide 30 mg twice weekly for 2 weeks, and azacitidine 100 mg daily on days 1-7) along with an aggressive antifungal treatment for pulmonary infection. Measures were taken to optimize his performance status. During the period of myelosuppression associated with VCA therapy, he suffered from grade II leukopenia and grade IV thrombocytopenia, for which platelet transfusion was performed (**Figure 2A**). The enlarged lymph nodes and soft tissue masses disappeared in CT scans following the first VCA course, indicative of a CR (**Figures 1A2, B2, C2, D2**). The pulmonary infection had resolved (**Figure 1E2**). On April 22, 2024, he received a second course. The period of BM suppression lasted for approximately 10 days, without any transfusion (**Figure 2B**). A positron emission tomography-computed tomography (PET-CT) performed on May 30, 2024, identified a soft tissue mass in the subcutaneous tissue and the tip of the sternum that exhibited increased ^18^F-FDG uptake (**Figures 1F, G**). Therefore, he underwent autologous CD19 CAR-T cell therapy with classic fludarabine/cyclophosphamide conditioning. A week later, the nodules on the right chest wall and the tip of the sternum completely disappeared. A subsequent PET-CT scan performed 10 weeks post-CD19 CAR-T cell therapy indicated complete metabolic remission (CMR). The patient remained in CR at the last follow-up. The disease timeline and treatment course of this patient is shown in **Figure 3**.

## 3 Discussion

To our knowledge, this is the first case of R/R B-ALL with MLL-r being treated with the combination of a BCL-2 inhibitor and epigenetic modulators, leading to CR. In this patient who presented with multiple extramedullary lesions, the VCA regimen significantly reduced the tumor burden and served as a successful bridge to CD19 CAR-T cell therapy.

The prognosis of patients with *MLL-AF4*–positive ALL is in general poor, with a 3-year overall survival of 24% following conventional chemotherapy. Although allo-HCT serves as a curative opportunity with a survival rate exceeding 60%, the 5-year overall survival among those who relapse post-transplantation is only 8% (6, 7). Further, the prognosis is poorer among patients with EMR where the mortality is 96.6% (28/29) (7). Recent breakthroughs in immunotherapies such as anti-CD19 CAR-T therapy have demonstrated promising outcomes with a response rate of 85–100% in B-ALL patients with *MLL-AF4* (8, 9). Studies have also underscored the significant activity of CAR-T therapy in EMD (10). However, this patient was ineligible for an immediate CAR-T therapy owing to poor performance status, severe pulmonary infections, rapid disease progression, which demanded 2-3 weeks for CAR-T cell preparation. A lower tumor burden at the time of CAR-T infusion is known to ameliorate long-term survival and reduce the risk of severe cytokine release syndrome and neurotoxicity (11). Therefore, a bridging therapy prior to CAR-T therapy was warranted to curb disease progression and lower tumor burden.

Common bridging therapies such as low-intensity chemotherapy and targeted agents such as inotuzumab ozogamicin (INO) and blinatumomab (12) were not suitable for this patient. Conventional chemotherapy is associated with a 64% failure rate in inducing remission among high-risk relapsed ALL patients, particularly those with KMT2A rearrangements or hypodiploidy (13). In EMD, the response rate with INO is 55–71% primarily in those with high CD22 expression (14, 15). The patient in our case, however, showed no CD22 expression. Although he was CD19 positive, the efficacy of CD3/CD19-directed bispecific T-cell engagers such as blinatumomab in EMD was poor. Numerous reports have indicated the persistent EMR among B-ALL patients treated with blinatumomab, plausibly owing to inadequate tissue penetration and T-cell recruitment to nonhematopoietic tissues (16–18). Therefore, we used the combination therapy exercising potential pharmacological synergy—VEN, CHI, and AZA—a targeted and immunomodulatory regimen with reduced toxicity.

The VEN, CHI, and AZA combination exerts synergistic antileukemic effects. Evidence suggests the high expression of Bcl-2 and MCL-1 in *MLL-AF4*-expressing cells (19, 20). These cells also exhibit extensive promoter hypermethylation, which leads to silencing of tumor suppressor genes and the consequent development of hematologic malignancies (21–23). Therefore, the Bcl-2 inhibitor VEN and the demethylating agent AZA exert therapeutic effects in patients with *MLL-AF4* fusion gene AZA enhances the antileukemic efficacy of VEN by lowering MCL-1, which reduces the leukemic resistance to VEN (24). Moreover, VEN and AZA synergistically eliminate leukemia stem cells by reducing amino acid uptake and disrupting the tricarboxylic acid cycle via electron transport chain complex II suppression (25–27). Histone deacetylases (HDACs) participate in the generation and functioning of *MLL* gene rearrangement (28, 29). Inhibitors of HDACs such as CHI activate endogenous *MLL* and consequently eliminate active products of *MLL-AF4*, thereby inhibiting the proliferation and viability of t(4;11) cells (30). CHI also enhances the antileukemic activity of VEN through MCL-1 and Bcl-xL downregulation and BIM upregulation (31). The combination of VEN and demethylating agents exerts significant efficacy, is well tolerated in AML and R/R B-ALL patients, and has been incorporated into clinical guidelines for elderly and/or unfit patients with AML (32, 33). Wang B.R. et al. reported promising results with this combination among three R/R AML patients (34). To our knowledge, there are no reports describing its use for B-ALL treatment.

Following patient’s consent, we initiated the VEN, CHI, and AZA combination for this relapsed patient post-haplo-HCT based on aforementioned research findings. Surprisingly, he achieved CR immediately following the first course. After two courses, the patient received CAR-T therapy and achieved CMR. Noteworthy, this is a chemotherapy-free regimen with mild and manageable adverse effects, primarily transient pancytopenia. It is particularly suitable for those with poor performance status and uncontrolled infectious complications.

In conclusion, the successful outcomes in this case serve as a preliminary indication of applicability of the VCA regimen in controlling rapid disease progression in B-ALL patients with *MLL-AF4* who experience EMR post allo-HCT, thereby providing an opportunity for subsequent treatment. However, conclusions drawn from a single case report are limited. Therefore, further clinical studies are needed to confirm the potential of the VCA regimen in B-ALL treatment.

## Data Availability

The raw data supporting the conclusions of this article will be made available by the authors, without undue reservation.
